# Role of Short Chain Fatty Acids to Counteract Inflammatory Stress and Mucus Production in Human Intestinal HT29-MTX-E12 Cells

**DOI:** 10.3390/foods11131983

**Published:** 2022-07-05

**Authors:** Carlotta Giromini, Antonella Baldi, Raffaella Rebucci, Davide Lanzoni, Martina Policardi, Tamil selvi Sundaram, Stig Purup

**Affiliations:** 1 Department of Veterinary and Animal Science, Università Degli Studi di Milano, Via dell’Università 6, 29600 Lodi, Italy; antonella.baldi@unimi.it (A.B.); raffaella.rebucci@unimi.it (R.R.); davide.lanzoni@unimi.it (D.L.); tamil.sundaram@unimit.it (T.s.S.); 2 CRC, Innovation for Well-Being and Environment, Università Degli Studi di Milano, 20122 Milano, Italy; 3 Diabetes Research Institute, IRCCS Ospedale San Raffaele, Via Olgettina, 60, 20132 Milano, Italy; martina.policardi@gmail.com; 4 Department of Animal Science, Aarhus University, DK-8830 Tjele, Denmark; stig.purup@anis.au.dk

**Keywords:** fatty acids, gut health, dietary fibre, mucus layer

## Abstract

Short chain fatty acids (SCFAs), especially butyrate (BUT), are known to promote intestinal health, but their role in the protection of intestinal barrier integrity is poorly characterized. The aim of the study was to set up an in vitro model of human colon epithelium using HT29-MTX-E12 cells to delineate the potential role of SCFAs under stress conditions. Accordingly, the HT29-MTX-E12 cells were differentiated for 42 days and subsequently exposed to dextran sulphate sodium (DSS). Further, the effects of BUT or its mixture with acetate and propionate (SCFAs-MIX) were tested to study proliferation, epithelial integrity and mucus production. The results showed that the concentration of 10% DSS for 24 h decreased the TEER about 50% compared to the control in HT29-MTX-E12 cells. The pre-treatment on HT29-MTX-E12 cells with BUT or SCFAs-MIX at specific concentrations significantly (*p* < 0.05) reduced the DSS-induced damage on epithelial cell integrity and permeability. Further, the treatment with specific concentrations of BUT and SCFAs-MIX for 24 h significantly promoted ZO-1, MUC2 and MUC5AC mRNA expression (*p* < 0.005). The present study demonstrated the suitability of HT29-MTX-E12 cells treated with DSS as an in vitro stress model of inflammatory bowel disease, which enabled us to understand the effect of bioactive SCFAs on the intestinal barrier.

## 1. Implications

The integrity of the gastrointestinal barrier plays an important role in human health but also in the performance and welfare of production animals. Short chain fatty acids play an essential role in maintaining intestinal health by affecting cell proliferation, epithelial integrity and mucus production. The present in vitro study supports the suitability of HT29-MTX-E12 cell line stressed with dextran sulphate sodium to be used as a viable model to investigate the effect of short chain fatty acids’ mechanism of action.

## 2. Introduction

Optimal gut health is crucially important in determining human and animal health, particularly in production animals due to the direct relationship between gastrointestinal tract (GIT) health and animal performance and welfare [1]. An important key component of the effective functionality of the intestinal barrier is represented by diet, combined with complex mechanisms of digestion and absorption of nutrients. The digestive system has different functions, of which the digestion of feed into small water-soluble molecules by enzymes and microbial activity is the foremost. Moreover, the GIT is the largest immune system, which acts as a biochemical or physical barrier between the host and the environment [1].

The EU-wide ban on the use of antibiotics as growth and health promoters in animal feed from 2006, together with the planned ban on the therapeutics use of zinc oxide in 2022, has shifted the research focus in search of reliable alternatives to antimicrobials (Regulation 1831/2003/EC and subsequent amendments). On 13 September 2018, the European Parliament adopted a resolution, “European One Health Action Plan against Antimicrobial Resistance”, that stresses the correct and prudent use of antimicrobials in order to limit the emergence of antimicrobial resistance in animal husbandry. Besides, it emphasizes the coordination between all regions in the European Union to undertake actions necessary for the prevention of antimicrobial resistance [2].

Generally, numerous complex mechanisms are involved in regulating the health and functioning of the GIT. Among these, diet, functional integrity of the gastrointestinal barrier, microbiota and an active immune system represent the main components controlling the gut health [3].

Factors such as the chemical composition, particle size and dietary formulation of fibrous fraction have a significant impact on the gastrointestinal functionality and physiology of animals. It has previously been demonstrated that a diet rich in fibre is able to reduce the risk of inflammatory diseases both in humans and animals [4,5].

Intake of fibrous compounds leads the GIT to a fermentative process by specific bacteria, which include the phyla of short chain fatty acids (SCFAs)-producing bacteria, such as *Bacteroidetes*, *Firmicutes* and *Actinobacteria* [6]. SCFAs are produced as a result of the fermentation of dietary fibres partially and non-digestible polysaccharides escaping digestion in the small intestine. Among the SCFAs, the most produced are, in particular, acetate (C2), propionate (C3) and butyrate (BUT) (C4). They play an important role in the maintenance of gut health and promotion of mucosal immune response in animals and humans. They are also involved in preventing the attachment and overgrowth of pathogens in the GIT [7,8]. Although the beneficial effects of organic acids, including the SCFAs on the intestinal health of production animals, have been widely demonstrated, the exact mode of action at the cellular and molecular level is still unknown [9,10,11].

The HT29-MTX-E12 cell line represents a suitable model for in vitro nutritional studies in both humans and mono-gastric animals; therefore, the aim of this study is to evaluate the effects of SCFAs on intestinal barrier integrity using HT29-MTX cells as an in vitro model of the human colonic epithelium. The specific aims of the study, after differentiating HT29-MTX cells up to 42 days in order to produce a stable amount of mucus needed to form a layer, are: (i) to test the effect of SCFAs in counteracting inflammatory stress induced by treatment with dextran sulphate sodium (DSS); (ii) to estimate the potential protective effect of SCFAs by assessment of changes in the mRNA expression of key genes involved in mucus secretion and tight junction integrity (MUC2 and MUC5AC, ZO-1 (zonula occludens)), analysed with real-time reverse transcription polymerase chain reaction (RT-PCR).

## 3. Materials and Methods

### 3.1. HT29-MTX-E12 Cell Culture and Treatments

The human mucus-secreting colorectal adenocarcinoma cell-line, HT29-MTX-E12, was kindly donated by Dr. Davide Brayden and Sam Mayer (UCD Conway Institute, Dublin, Ireland). The cells were maintained in Dulbecco’s modified Eagle Medium (high glucose DMEM, Euroclone, Wetherby, UK) supplemented with GlutaMAX^®^ (Gibco, Paisley, UK), 10% of heat inactivated foetal bovine serum (Euroclone). The cells were maintained at 37 °C in a humidified atmosphere providing 5% CO_2_. The cells were passaged at 70% to 80% confluency using 0.05% of Trypsin-EDTA (Euroclone) and seeded at the density of 3 × 10^5^ per cell culture insert place in the 12-well plate. After seeding, the cells were maintained in culture until full differentiation of 42 days. At day 43, cells were treated apically with 10% of DSS (sterile filtered, 40 kDa molecular weight, Sigma-Aldrich, St. Louis, CO, USA) for 2 h. Further, cells were treated with 1 mM, 0.1 mM and 10 mM of BUT or SCFAs-MIX (sodium propionate, acetate BUT) for 1, 6 and 24 h.

A negative (DMEM with 2% FBS) and a positive control (DSS 10% without FBS) were included in the study.

### 3.2. Epithelial Integrity

The integrity of the cell monolayer was measured with transepithelial electrical resistance (TEER) during the 42 days differentiation, before and after treatment with DSS, BUT and SCFAs-MIX. TEER was measured by using Millicell-ERS volt-ohm meter combined with a MERSSTX03 electrode (Millipore, Hellerup, Denmark) according to the manufacture’s guidelines. Presented values were corrected for background resistance from culture inserts and are given Ω (resistance) × cm^2^ (surface area of the cell layer) of a treatment/Ω (resistance) × cm^2^ (surface area of the cell layer) of control (DSS 10%, *w*/*o* FBS).

### 3.3. Paracellular Tracer Flux Assay

Fluorescein isothiocyanate-dextran (4.000 kDa molecular weight: Sigma-Aldrich, FITC-dextran) was used to calculate the permeability across epithelial monolayer after treatments. In particular, 1 mL of FITC-dextran was added apically at a concentration of 2.2 mg/mL in DMEM without FCS and incubated for 1 h at 37 °C. The flux of FITC-dextran across the HT29-MTX cell monolayer was measured by collecting samples in the basolateral compartment. Fluorescence was recorded on an EnVision 2103 Multilabe Reader (PerkinElmer Inc. Waltham, MA, USA) with excitation and emission wavelengths set at 490 nm and 520 nm, respectively. The supernatants from the apical compartment collected before addition of FITC-dextran were tested with lactate dehydrogenase (LDH) assay for evaluation of the integrity of the membrane.

### 3.4. Membrane Stability

The membrane stability of HT29-MTX cells was tested on 42 days cell supernatant with LDH assay (CytoTox 96, Promega, Madison, WI, USA), as reported in the manufacturer’s instruction, after treatment with DSS, BUT or SCFAs-MIX.

### 3.5. Mucus Production-RT-PCR

The HT29-MTX-E12 cells were collected in RNAlater and stored at −80° C. Total RNA was isolated and purified according to the Nucleospin RNA II manual (machery-Nagel, Duren, Germany) according to the manufacturer’s protocol. The mRNA (50 ng) was reverse-transcribed using the iScript cDNA synthesis Kit (BioRad Laboratories, CA, USA). The cDNA samples were diluted in 1:6 ration and the RT-PCR assay was carried out using the real-time fluorescence method (Strategene MX3000p). MUC5AC, MUC2 and ZO-1 genes were amplified by RT-PCR with primer sequences that were previously published in Martinez-Maqueda et al., (2012) and Nielsen et al., (2018) (Table 1); cyclophilin and β-actin were included as reference genes. The primers have been checked for specificity in BLAST before the RT-PCR experiments [12,13].

Each reaction tube contained 2X SYBR Green real-time PCR Master Mix, gene-specific forward and reverse primers and the cDNA (1 µL). The master mix included Maxima^®^ Hot Start Taq DNA polymerase, dNTPs in an optimized PCR buffer and SYBR^®^ Green I dye supplemented with ROX passive reference dye. All reactions were analysed under the same conditions and normalized to the ROX reference dye to correct any fluctuations in the reading due to evaporation phenomena. The samples were tested in triplicate and the non-reverse-transcribed controls and no-template controls were included in the assays. The thermal profile began 4 min at 95 °C for 30 s and 60 °C for 1 min. Relative quantification was performed, and the values were normalized to the internal reference gene cyclophilin. Two internal controls, cyclophilin and β-actin genes, were tested as endogenous genes.

### 3.6. Statistics

The data from cell viability, TEER and FITC-dextran studies were analysed by one-way analysis of variance (ANOVA) using GLM procedures of Graph Pad Prism Version 9.0). Results are expressed as mean ± SEM. Significant difference was set at a *p*-value less than 0.05.

For gene expression analysis, the comparative CT method was used to determine the fold changes in gene expression, which were calculated using the threshold method (2^−∆∆CT^) [14]. A two-way ANOVA was performed on cell metabolic activity and gene expression data, and the means were compared using Tukey’s test. Differences between means were considered statistically significant at *p* < 0.05 (*), *p* < 0.001 (**) and *p* < 0.0001 (***).

All graphical representations were made using Graph Pad Prism software.

## 4. Results

### 4.1. Cell Integrity

#### 4.1.1. TEER

**Figure 1:**
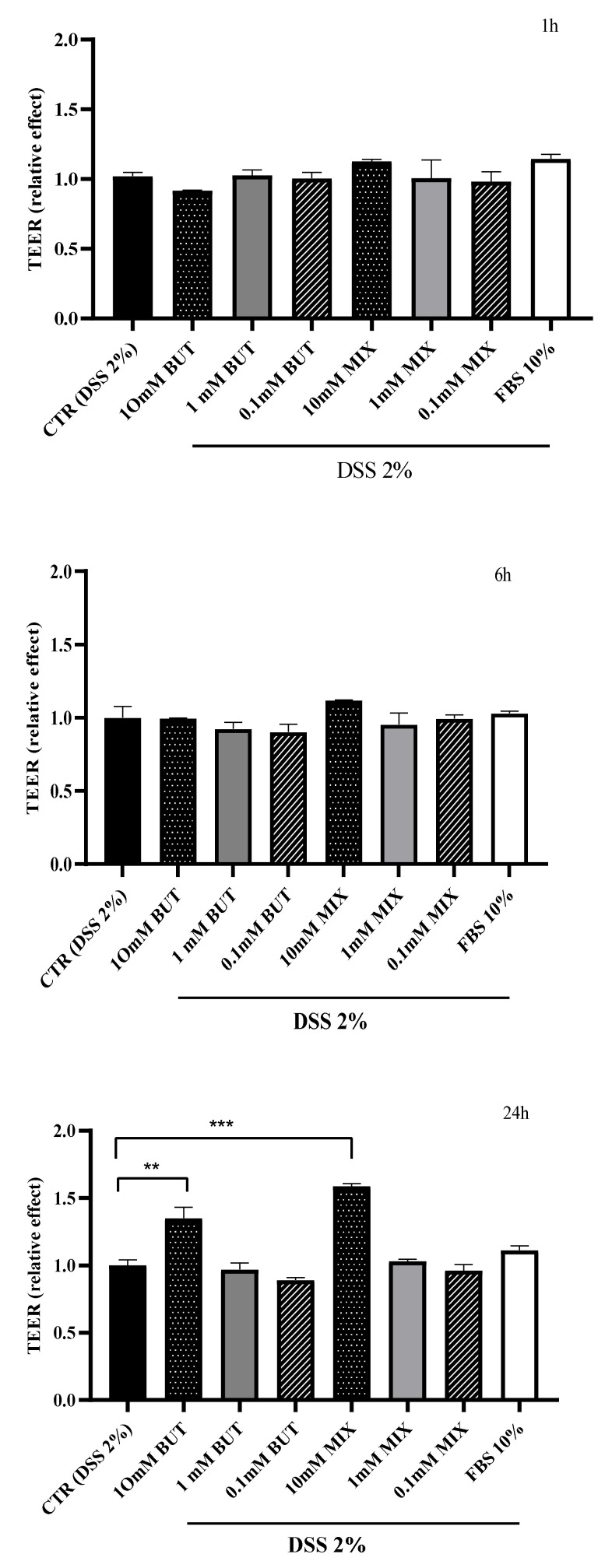
Effect of butyrate (BUT) and short chain fatty acids mix SCFAs-MIX) on the transepithelial electrical resistance (TEER) of differentiated HT29-MTX-E12 cells exposed to DSS stress. Cells were treated apically with DSS for 24 h and further maintained with BUT or SCFAs-MIX for 1, 6 or 24 h, 10% fetal bovine serum (FBS 10%). Data are expressed as mean values ± SEM relative to control (cells treated with dextran sulphate sodium (DSS) for 24 h and further maintained in basal medium for 1, 6 or 24 h). Data are mean ± SEM of three independent experiments. Differences between means were considered statistically significant at *p* < 0.001 (**) and *p* < 0.0001 (***).

The treatment with DSS decreased the integrity of the HT29-MTX-E12 monolayer. This effect was demonstrated by the reduction in the TEER value after 24 h of treatment with DSS, from 2028 ± 41 Ω·cm^2^ to 1017 ± 26 Ω·cm^2^. Further, HT29-MTX-E12 cells were treated with DSS and with three different concentrations of BUT and SCFAs-MIX (0.1, 1 and 10 mM) or with FBS (10%) for 1, 6 and 24 h. As shown in Figure 1, the cytotoxic effect induced by DSS was significantly (*p* < 0.05) reduced after 24 h of treatment with 10 mM of BUT and SCFAs-MIX.

#### 4.1.2. FITC

**Figure 2:**
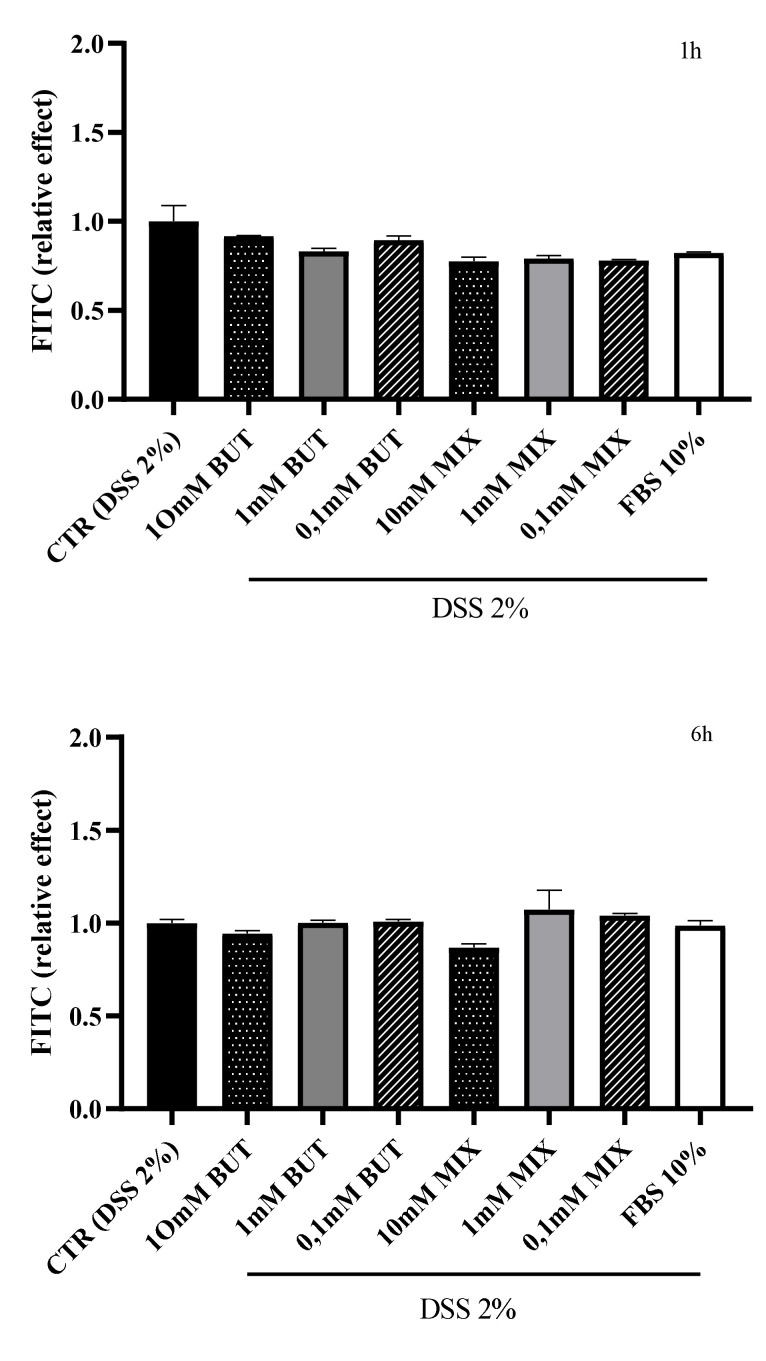

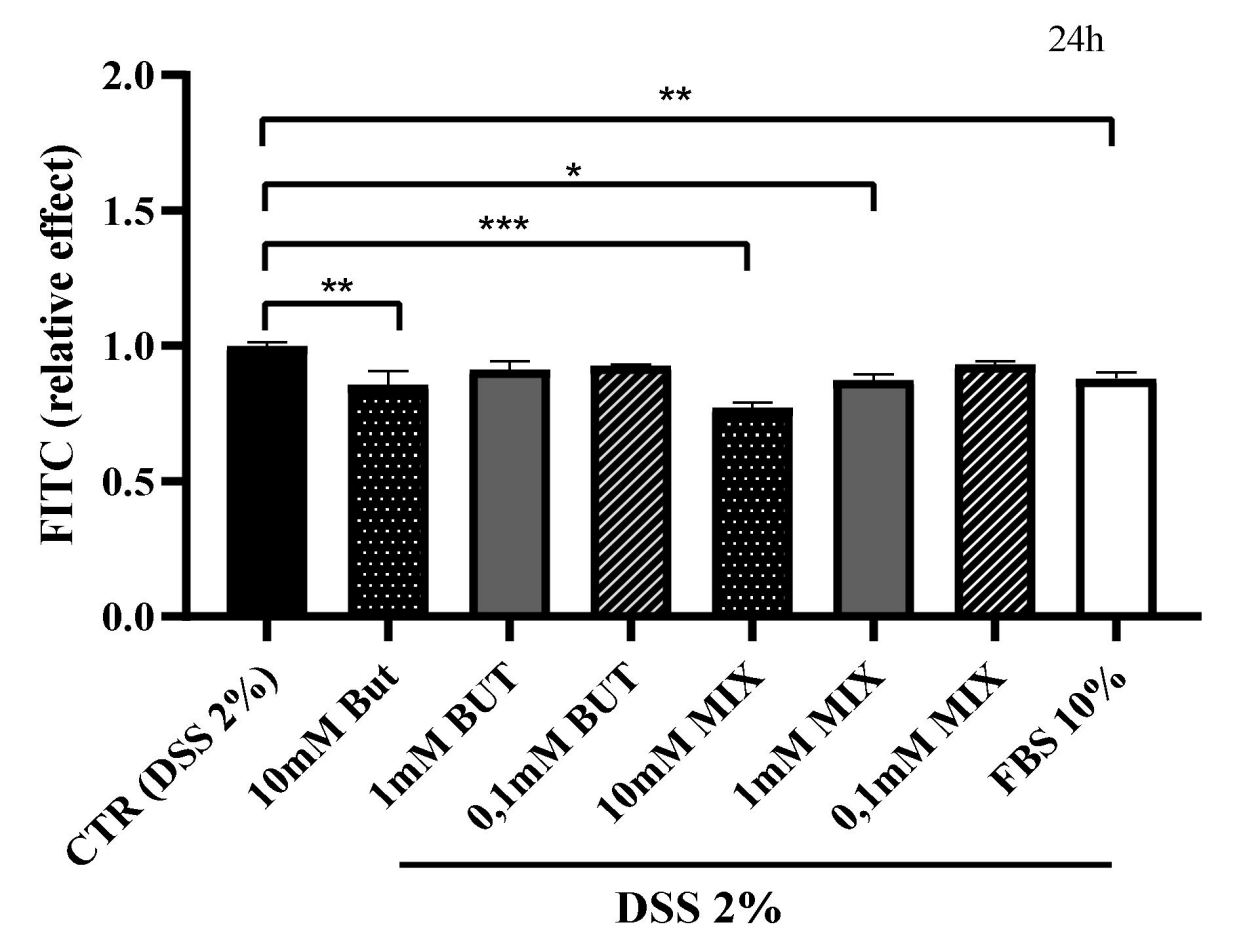
Effect of butyrate (BUT) and short chain fatty acids mix (SCFAs-MIX) on the fluorescein isothiocyanate (FITC-dextran) of differentiated HT29-E12 cells. Cells were treated apically with DSS for 24 h and further maintained with BUT and SCFAs-MIX for 1, 6 or 24 h, 10% fetal bovine serum (FBS). Data are expressed as mean values ± SEM relative to control (cells treated with DSS for 24 h and further maintained in basal medium for 1, 6 or 24 h). Data are mean ± SEM of three independent experiments. Differences between means were considered statistically significant at *p* < 0.05 (*), *p* < 0.001 (**) and *p* < 0.0001 (***).

The epithelial integrity was subsequently evaluated measuring the FITC-dextran values after 1, 6 and 24 h of treatment with BUT and SCFAs-MIX at three different concentrations (10 mM, 1mM and 0.1 mM) and with FBS (10%). The results are shown in Figure 2. No significant differences were observed after 1 and 6 h of treatment. After 24 h of treatment with 10 mM of BUT, the FITC-dextran was significantly reduced (*p* < 0.05). In particular, 10 mM BUT, 10 mM and 1 mM SCFAs-MIX were able to significantly (*p* < 0.05) counteract the DSS-induced cellular damage. Treatment with 10% FBS had a positive effect in counteracting the epithelial damage after 24 h, even if to a lesser extent compared with 10 mM SCFAs-MIX.

### 4.2. Membrane Stability

**Figure 3:**
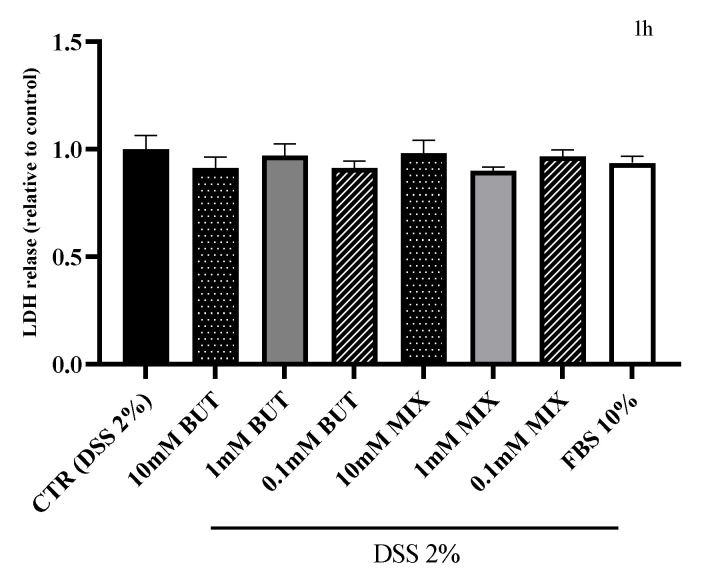

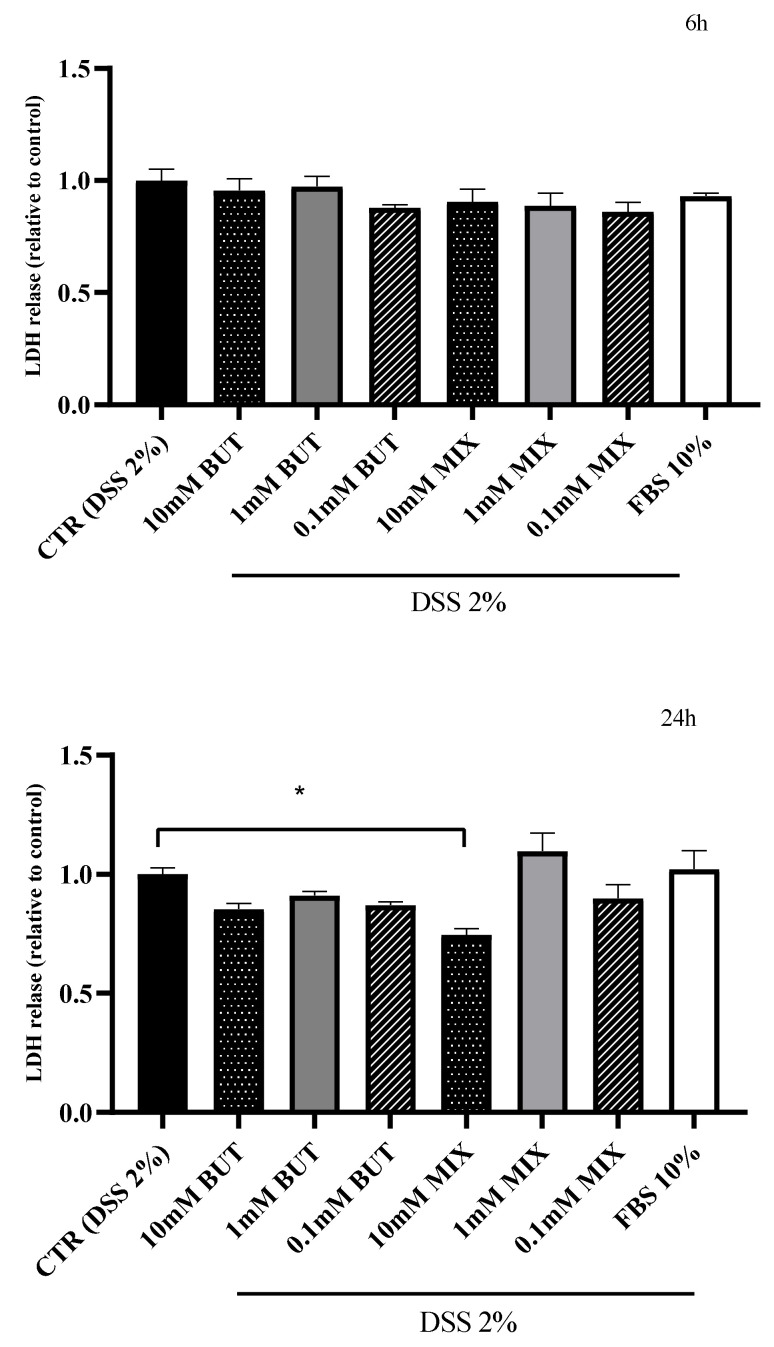
Effect of butyrate (BUT) and short chain fatty acids mix SCFAs-MIX on the LDH release of differentiated HT29-MTX-E12 cells. Cells were treated apically with BUT and SCFAs-MIX for 1, 6 or 24 h, 10% (FBS) and compared with control (cells treated with DSS for 24 h and further maintained in 10% FBS for 1, 6 or 24 h). Data are mean ± SEM of three independent experiments (*n* = 3). Differences between means were considered statistically significant at *p* < 0.05 (*).

In order to evaluate the epithelial integrity, the release of LDH was tested after 1, 6 and 24 h of treatment with BUT and SCFAs-MIX at three different concentrations (10 mM, 1 mM and 0.1 mM) and with FBS (10%). The results are shown in Figure 3. Treatment with 10 mM SCFAs-MIX significantly reduced the LDH release compared with the control after 24 h of treatment.

### 4.3. Gene Expression

**Figure 4:**
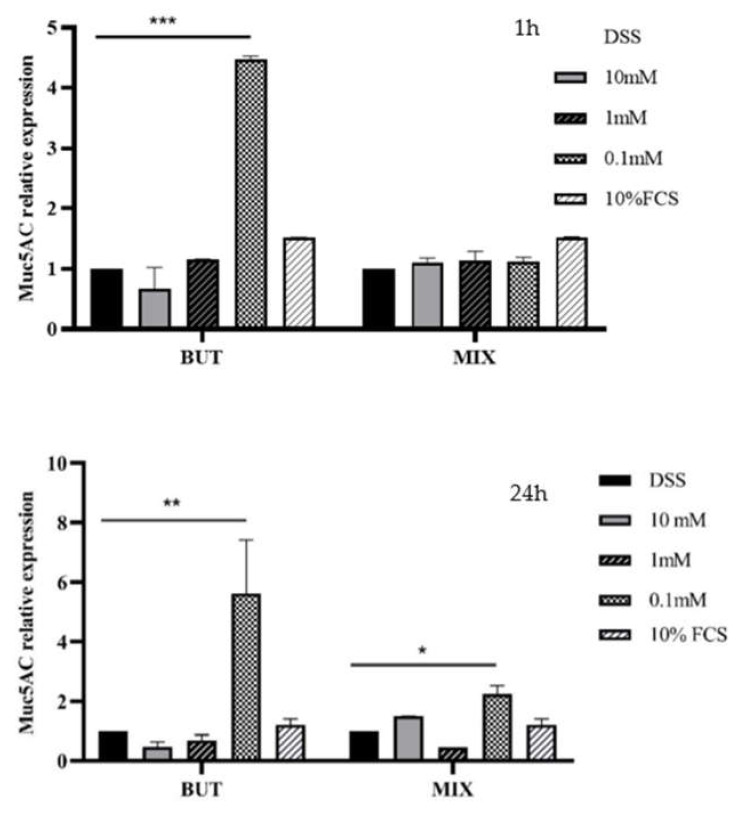
MUC5AC relative expression after 1 and 24 h treatment. Data are from three independent experiments. Data are mean ± SEM of three independent experiments (*n* = 3). Differences between means were considered statistically significant at *p* < 0.05 (*), *p* < 0.001 (**) and *p* < 0.0001 (***).

**Figure 5:**
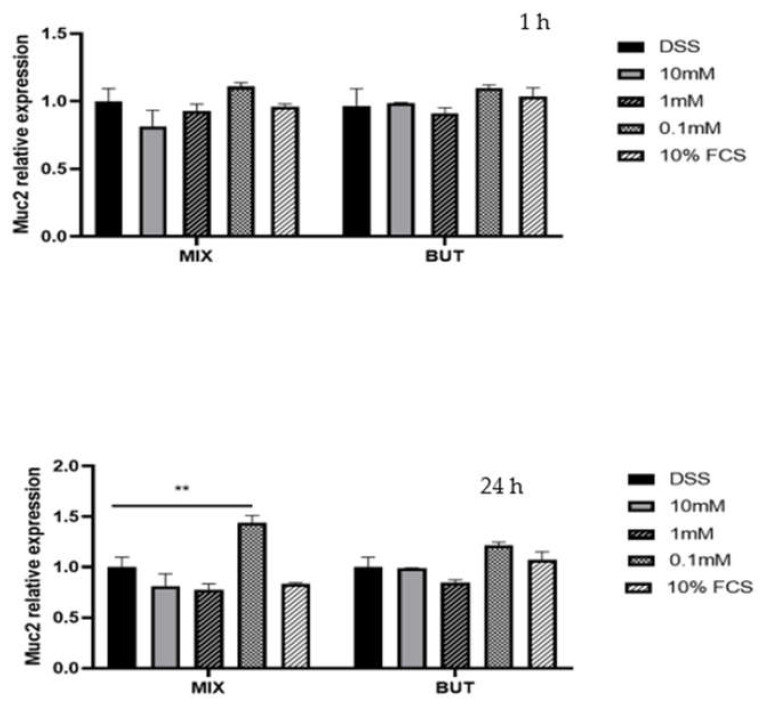
MUC relative expression after 1 and 24 h treatment. Data are from three independent experiments. Data are mean ± SEM of three independent experiments (*n* = 3). Differences between means were considered statistically significant at *p* < 0.001 (**).

**Figure 6:**
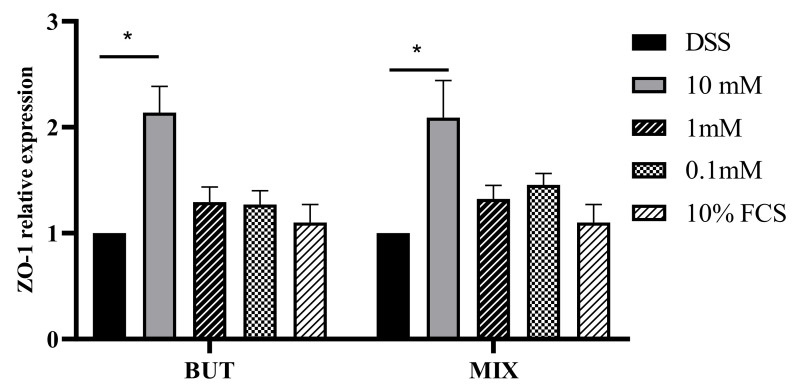
ZO-1 relative expression after 24 h treatment. Data are from three independent experiments. Data are mean ± SEM of three independent experiments (*n* = 3). Differences between means were considered statistically significant at *p* < 0.05 (*).

HT29-MTX-E12 cells were treated for 1 and 24 h in the presence of BUT and SCFAs-MIX at 10, 1, 0.1 mM. Specific concentration modulated the expression of mucins MUC5AC, MUC2 and ZO-1 in HT29-MTX-E12 cells. In particular, BUT and SCFAs-MIX increased (*p* < 0.05) the expression of MUC5AC and MUC2 mRNA at 0.1 mM compared with the cells treated with DSS (10%) (Figure 4 and Figure 5). The expression of ZO-1 mRNA was increased by BUT and SCFAs-MIX at 10 mM after 24 h treatment (Figure 6).

## 5. Discussion

Our study demonstrated the beneficial effect of SCFAs on the intestinal epithelial barrier function in vitro. We measured the intestinal epithelial barrier function, such as the changes in TEER and flux and FITC-dextran across the HT29-MTX-E12 cell monolayer. TEER is a non-invasive and label-free method used to measure the electrical resistance across a cell monolayer. The resistance measure reflects both the transcellular and paracellular integrity. We used the TEER method to monitor cell growth and differentiation up to 42 days [15], whereas the transport of the FITC-dextran across the cell monolayer reflects the selective permeability, transport capacity as well as the pore size of the tight junctions [16]. Therefore, often but not necessarily, both the TEER and FITC-dextran display a parallel endpoint in the intestinal permeability assessment.

At day 42, the cells were exposed to 10% DSS for 24 h to stimulate the intestinal barrier damage that occurs during an inflammatory bowel disease.

Inflammatory diseases are derived from a complex interaction between several factors, among which one of the most important is represented by diet. During inflammation, the absorption rate of essential nutrients decreases, and the mucosal immune system of the gut is continuously affected. This can disrupt the digestion and nutrition absorption, leading to inflammatory diseases, which, consequently, lowers the animal performance and welfare [1,17].

The use of DSS as an inflammatory stress inducer has been previously established [18]. Previously, the harmful effects induced by DSS on the integrity of the intestinal barrier were demonstrated in both in vivo (in pigs: Ref. [19]) and in vitro [13] studies. In this study, a 40-kDa DSS was used. The molecular weight of DSS is crucial for inducing an inflammatory response [20]. DSS with a molecular weight of 36 to 50 kDa is commonly used to induce bowel inflammation in animal models. In this study, the extent of the cellular damage was evaluated measuring the TEER values. In agreement with the study conducted in Ref. [13], the treatment with 10% DSS for 24 h was able to decrease the TEER values by almost 50%.

Our previous study [13] demonstrated the ability of sodium-BUT to improve barrier function in DSS-damaged HT29-MTX cells as measured by TEER and FITC-dextran methods. Based on the obtained results, we tested the ability of the combination of major SCFAs on this cell line as fatty acids are physiologically present in the gut as a mixture.

We found that SCFAs at specific concentrations were able to counteract the cytotoxic effect induced by DSS. Our results showed that the capability of BUT to increase the TEER values was in accordance with Ref. [21]. They demonstrated that colonocytes from rats with DSS-induced colitis, to which BUT had been administered, showed significantly higher TEER (115 ± 2.0 µohm∙cm^−2^∙h^−1^) values compared to the control (rats with DSS-induced colitis that did not receive butyrate administration) (60.2 ± 0.7 µohm∙cm^−2^∙h^−1^). Moreover, the beneficial effect of BUT by oral administration on intestinal morphology and barrier function in pigs was investigated by Wang et al. (2018) [22]. They demonstrated that a diet containing 450 mg/Kg sodium-BUT was able to significantly increase the TEER (61.68 ± 6.37 Ω∙cm^2^) compared with a diet without BUT supplementation (51.52 ± 5.57 Ω∙cm^2^). Changes in the TEER values from our results showed that the synergistic action of a mix of SCFAs-MIX was also able to reduce the DSS-induced cellular damage after 1 and 24 h of the treatments. Our results are also in accordance with an earlier study on Caco-2 cells by the authors of Ref. [23], who demonstrated that 2 mM BUT was able to promote the intestinal barrier integrity. Further, the FITC-dextran measurements showed that BUT and SCFAs-MIX at different concentrations were able to reduce the paracellular permeability of HT29-MTX cells. These observations were in agreement with Nielsen et al. (2018) [13], who demonstrated that 1–10 mM BUT application was able to significantly improve the paracellular permeability and epithelial integrity compared to the DSS control. In our study, generally, SCFAs-MIX showed greater effects than BUT in increasing paracellular permeability. Following, the authors of Ref. [24] tested the effects of SCFAs on the intestinal permeability of four-week-old female mice with 5-fluoroacil-induced mucositis. They demonstrated that BUT (9 mM) was able to reduce the intestinal permeability to certain levels that were closer to the control groups (with no mucositis induction) while higher than SCFAs combination (35 mM of acetate, 15 mM of propionate and 9 mM of BUT).

The damage to intestinal epithelial barrier function due to DSS may reduce the thickness of the mucus layer. The mucus layer on the intestinal epithelium acts as the first line of host-defence that forms a physical and chemical barrier against various pathogenic stimuli. Optimal integrity and function of the mucus layer are crucial in ensuring proper absorption of nutrients and preventing the attachment and growth of pathogens to the intestinal mucosa. The intestinal epithelium is formed by different types of intestinal epithelial cells, including the goblet cells, whose main role is to secrete mucus. The ability of these cells to produce mucus can be mimicked in in vitro conditions using the mucus-secreting cell line, such as the human colon adenocarcinoma HT29-MTX cell line [25]. These cells represent a viable alternative to the in vivo studies carried out in the mono-gastric animals’ intestinal environment [8,12,25,26]. In this study, a stable cell culture of HT29-MTX cells was used. The cells were cultured for 42 days on transwell inserts to ensure an adequate production of mucus.

HT29-MTX cells represent a model of the goblet cells in the intestinal barrier, which secrete a mucus layer upon cell differentiation. However, the use of this model comprises up to 80% goblet-like cell [25], whereas, in in vivo experiments, the proportion of goblet cells increases gradually from 4% to 20% in the region spanning the duodenum to colon.

The evaluation of the gene expression showed that SCFAs at the lowest concentration tested (0.1 mM) were able to significantly increase the MUC5AC and MUC2 expression after 1 and 24 h. In a previous study [27], it was demonstrated that MUC5AC gene expression was significantly decreased both by BUT (2 mM and 5 mM) and SCFAs-MIX (2 mM BUT, 2 mM propionate and 6 mM acetate) after 24 h [27]. Nielsen at al., (2018) found that BUT 1–5 mM maintained the MUC5AC expression at levels that were closer to those of the control group (cells treated with DSS and 10% FBS in deoxyvalenol-treated HT29-MTX-E12 cells), while BUT 0.1 and 10 mM significantly decreased the MUC5AC expression [13]. These results confirm that the effects of BUT and SCFAs-MIX on MUC genes expression are dose-dependent, as reported in previous studies [13,28]; therefore, a concentration below 0.1 mM needs also to be considered to enhance the epithelial barrier function.

Tight junctions are one of the main models of cell–cell adhesion that play a fundamental role in the intestinal barrier function, preventing bacteria, endotoxins and toxic macromolecules from crossing the epithelium. These are organized by specific interaction between the transmembrane proteins and intracellular proteins, one of them ZO-1 [29].

As reported earlier, reduction in ZO-1 is closely linked to decreased integrity and increased permeability of the epithelium [30]. These studies corroborate our data; in fact, treatment with BUT and SCFAs-MIX at 10 mM after 24 h led to an increase in the gene expression level of ZO-1. The same concentration and time point resulted in an improvement in epithelial structure, demonstrating once again the positive correlation that exists between ZO-1 and epithelial integrity.

In order to enhance animals’ performance and reduce the use of antimicrobial substances, it is necessary to consider new nutritional strategies, including those that can improve the production of colonic SCFAs quantitatively and qualitatively. This goal can be achieved by modulating the quantity and quality of the dietary fibrous compounds and their form and size by administering SCFAs directly or even by administering probiotics as feed additives. A diet rich in fibre represents the first step in ensuring a substantial production of SCFAs by colonic bacteria and overall intestinal health. However, in the present study and in accordance with previous publications, it was found that low concentrations of SCFAs promote intestinal barrier function, while high concentrations have no beneficial effects and may even disrupt the barrier functions [31]. Thus, fibres with slow but steady SCFAs production could be beneficial in vivo compared to quickly fermented fibres that localize high SCFAs concentration. These nutritional strategies may represent preventive strategies to limit the gastro-intestinal inflammatory problems in farm animals, which, in turn, may lead to a reduction in the use of antimicrobial substances to control intestinal infections. This is in line with the EU policy, which imposes to study new solutions to reduce the use of synthetic antimicrobials as much as possible, especially in food-producing animals. In fact, since the EU-wide ban on the use of antibiotics as growth and health promoters in animal feed came into force (2006), and after the CVMP demand to control the environmental presence of zinc oxide (2017), it became necessary to develop alternative strategies to antimicrobials. The increase in SCFAs production could lead to an improvement in animal production and welfare.

Although the HT29-MTX-E12 cell line represents a suitable model for in vitro nutritional studies both for human and mono-gastric animals, to characterize the SCFAs mechanism of action at the intestinal level, it would be necessary to set up complex models considering the active participation of the intestinal microbiota, which has a key role in the processes of digestion and absorption of nutrients, the immune system response and in the overall organism’s defence, before progressing with in vivo studies.

## 6. Conclusions

In conclusion, this study demonstrated that the SCFAs, at specific concentrations, were able to partially restore the DSS-induced damage on HT29-MTX-E12 cells. Specifically, the SCFAs promoted epithelial integrity and stimulated the expression of genes involved in mucin production. Unfortunately, the impact of SCFAs in the microbiome was not assessed in the present study, but it would be worthwhile to investigate how these changes could influence our results.

## Figures and Tables

**Table 1:** Primer’s sequences.

Gene	Primers	References
MUC5AC	5′-CGACCTGTGCTGTGTACCAT-3′	[12]
	3′-CCACCTCGGTGTAGCTGAA-5′	
MUC2	5′-ACCCCAAGCCCTTCTACGAG-3′	[13]
	3′-GAGTGGATGCCGTTGATGGT-5′	
ZO-1	5′-AGAGGTGTTCCGTGTTGTGGA-3	[13]
	3′-GGCTGAGCGGACAAATCCT-5′	
B-actin	5′-CTTCCTGGGCATGGAGTC-3′	[12]
	3′-GCAATGATCTTGATCTTCATTGTG-5′	
Cyclophillin	5′-TCCTAAAGCATACGGGTCCTGGCAT-3′	[12]
	3′-CGCTCCATGGCCTCCACAATATTCA5′	

## Data Availability

The data presented in this study are available on request from the corresponding author.
